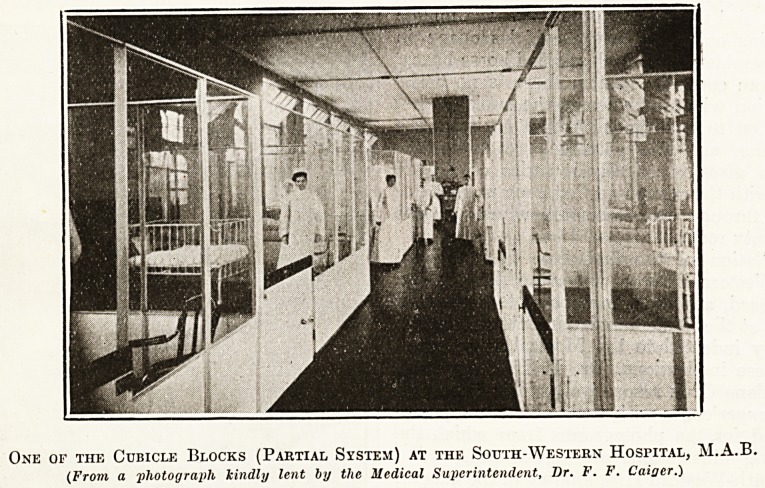# Preventive Methods in Provincial Institutions

**Published:** 1912-08-24

**Authors:** A. Knyvett Gordon

**Affiliations:** formerly Medical Superintendent of Monsall Hospital and Lecturer on Infectious Diseases in the University of Manchester.


### SOME FEVER HOSPITALS AND THEIR WORK.*

### V.—Preventive Methods in Provincial Institutions.

By A. KNYVETT GORDON, M.B. Cantab., formerly Medical Superintendent of Monsall Hospital and Lecturer on Infectious Diseases in the University of Manchester.

We now come to another method of prevention of cross-infection, which aims at isolating each patient either partially or completely by glass partitions, so that he is enclosed in a cell or cubicle.

The first point which must be borne in mind about a cubicle system is that it applies to an isolation ward only; so far as I know, it has never been suggested that all the general wards of a large hospital should be built on this plan: it must not, therefore, be confused with the barrier system which is intended to be applicable to any or every ward.

Then there are two kinds of cubicles, which differ, however, more in degree than in kind. In the partial system the partitions do not extend to the ceiling of the ward, and there is an aisle down the middle of the building on to which the cubicles open: in fact they resemble a row of glass-sided horse boxes.

In the more complete system there is no aërial communication whatever between the cubicles, but each opens on to a verandah which encircles the whole pavilion and the partitions reach to the ceiling; this gives practically a series of private rooms, but with the advantage that each patient can be watched through an observation window in the kitchen or duty room.

An installation of the first type is in use at the South-Western Hospital under the Metropolitan Asylums Board, and the more complete separation is employed at the Enfield and Edmonton Hospital. I am greatly indebted to the Medical Superintendents of these institutions, Dr. F. F. Caiger and Dr. R. Haldane Cook respectively, for information as to their experiences with their different types of cubicles, and for the photographs from which the accompanying illustrations are reproduced.

At the South-Western Hospital two of the general wards were converted into a cubicle system by erecting a series of glass partitions framed in iron reaching to a height of 7 feet above the floor level, leaving a passage about 5 feet wide down the centre of the ward: the doors, however, of the cubicles are only 3 feet 6 inches in height, the space above being unenclosed. The ward being 14 feet high, a space of 7 feet is left above each cubicle; so that, although each cubicle has a window to itself, it is also "ventilated" to a certain extent into the air of the general ward, four open fireplaces serving partly as extractors.

The precautions against infection by the staff are very complete. Separate overalls are provided and kept within each cubicle, and there is also a fixed washbasin with hot and cold water supply to a rose operated by a pedal tap in each compartment for the cleansing of the hands of the nurse on entering and leaving the compartment. Separate utensils are provided within the cubicle for each patient, and are sterilised by boiling, pretty much as in the barrier system previously described: a portable bath is used which can be taken into each cubicle. Very full instructions are given to the nurses as regards asepsis, not only in treatment, but in the serving of meals, etc., and it is impressed upon them that the avoidance of infection depends upon the carrying out of these directions, and not entirely on the separation afforded by the glass partitions.

As regards results, the cubicle ward was used for practically every kind of infection (except smallpox), and also for patients who were not suffering from any infectious disease at all, and it was found that 33 out of 1,069 patients contracted infectious diseases other than those for which they were

The Cubicle Block at the Enfield and Edmonton Isolation Hospital.

*(From a photograph kindly lent by the Medical Superintendent, Dr. R. Haldane Cook.)*

admitted. Practically the only infections transmitted were scarlet fever, measles, and chicken-pox, while no patient contracted either whooping-cough, mumps, influenza, or enteric fever, and only two rubella and one diphtheria. Dr. Caiger's experience on this point is worth quoting. He says: "As the result of four years' continuous observation . . . I am of opinion that cubicles . . . are unsuited for the treatment of smallpox, chicken-pox, and measles during the acute stage of the illness, and there is apparently some risk in the case of septic scarlet fever. On the other hand, scarlet fever if not of a septic type, diphtheria, measles in the most eruptive stage, rubella, whooping-cough, mumps, influenza, and probably typhus fever can

* Previous articles appeared in The Hospital of July 13, 20, Aug. 10, 17.

be treated without much fear of accident. Success or failure in these diseases is mainly a question of aseptic nursing."

In the other plan the separation is much more complete. The ward is entirely divided into a series of glass-sided rooms by partitions which extend to the ceiling, and the opening of each cell is on to a glazed verandah which runs right round the block, and along which the nurses walk. Each cell has a window and a hot-water radiator to itself. Cross ventilation is effected by means of the window, door, and ventilators (under and over each bed) on the outside walls, and on the side opposite the outside wall of the cubicle air is admitted through ventilators fitted to air-ducts which are carried under the wall of the adjoining cubicle to the outside of the building on the opposite side, and finished off with an air inlet grating. A foul-air outlet or extractor is fitted in the ceiling of each cubicle and carried through the roof by separate shaft to the open air. There is thus no aërial communication whatever between the compartments.

Immediately outside the door on the verandah is a lavatory basin with hot and cold water supply—one for each cell, and also a hook on which coats for the doctor and nurse are hung, each marked for the patient for whom it is to be used. The nurses duty room is entered on one side of the building by a door opening direct on to the verandah, and on the other side from the verandah by a passage having store-rooms on each side. An observation window is provided on each side of the duty room from which the interior of each cubicle can be seen.

At each end of the building, but entirely separate therefrom, are the w.c.s, rooms for hospital sinks, and a room for a portable bath, which is wheeled into each cubicle as required. Each patient has his own separate requisites and utensils, which are kept in his cell. There are twelve cells in the ward, six on each side of the duty room. Wards of this type are in use at Walthamstow and at the Enfield and Edmonton Joint Hospital, which, I understand, is a copy of the original building at Walthamstow. This system gives the most complete separation possible, and each patient is practically in his own private house, but in addition everything possible is done to avoid carrying infection by the nurses in going from one patient to another. In practice the system has worked excellently as regards results, for all kinds of mixed infections have been safely treated in the one block. No cross infection whatever has occurred so far.

The only point is whether structural separation is necessary. At Fazakerley, by the system of bed isolation previously described, the same results have been obtained without any structural separation, but the experiment has been made there with specially skilled nursing. The point, therefore, is whether the cubicle system minimises any risk that might occur from carelessness in disinfection on the part of the nurses, or, in other words, whether it allows less skilled or less expensive nursing to be employed. One would imagine that this might be the case, but it is impossible to make a dogmatic statement. There is always the idea that cubicles tend to impart a false sense of security in the mind of a partially trained or careless nurse.

One of the Cubicle Blocks (Partial System) at the South-Western Hospital, M.A.B.
*(From a photograph kindly lent by the Medical Superintendent, Dr. F. F. Caiger.)*

### Open-Air Nursing at Nottingham.

We come now to another point in the treatment of scarlet fever—namely, the practice that appears to be gaining ground considerably of nursing patients in the open air. Much work has been done on this subject by Dr. P. Boobbyer, Medical Officer of Health for Nottingham, at the isolation hospital for that city, and I am indebted to him for much valuable information on the whole subject. I have also had the advantage of personally visiting the hospital under his guidance and of seeing the conditions which obtain there.

The attempt was first made in 1889, and in 1895 it became a regular practice at the Nottingham Hospital to treat severe cases of scarlet fever in the acute stage by continuous exposure in the open air. The beds are placed in the corridors of the hospital, which are covered in by a roof only, and are exposed at the sides. The patient is sheltered by sail-cloth curtains in the very bad weather.

When the patient is first brought into the open air care is taken that he is kept warmly clad by means of extra blankets, and it is sometimes necessary to fasten the coverings to the bed itself. Hot-water bottles are generally necessary in winter. Tolerance is very soon established, and Dr. Boobbyer has found that septic cases especially do very well under these conditions, and that the results have been far better than they were before the system was in vogue, the scarlatinal death-rate, for instance, having been exactly halved. It has also been found that complications such as pneumonia and nephritis, which might perhaps have been expected to show themselves if the treatment were erroneous, have actually been less frequent than formerly. In 1903 one of the blocks was converted into an open-air pavilion with external verandahs, with the result that cross infection, which had been rather prevalent previously, practically ceased. The great difficulty was the attitude of the nurses and of the relatives of the patients. When the open-air pavilion was started the nurses protested strongly against the exposure involved (to the staff) in attending to the patients therein, especially in winter, but a complete change has taken place, and there is now no pavilion so popular amongst the nurses themselves. Similarly, while at first the relatives of the patients protested against the exposure of their children, it is now a frequent experience for them to ask that they shall be treated in the open, rather than in the closed wards. The example of Nottingham is being gradually copied in other hospitals, and there seems to be no doubt that, providing that the patient is kept warm by adequate coverings to the bed, a tolerance is quickly established, which soon gives place to a preference for the open air. When it is desired to use the old-fashioned type of wooden buildings, these can be made more safe as regards the risk of cross infection by converting them into "open-air" wards.

## Figures and Tables

**Figure f1:**
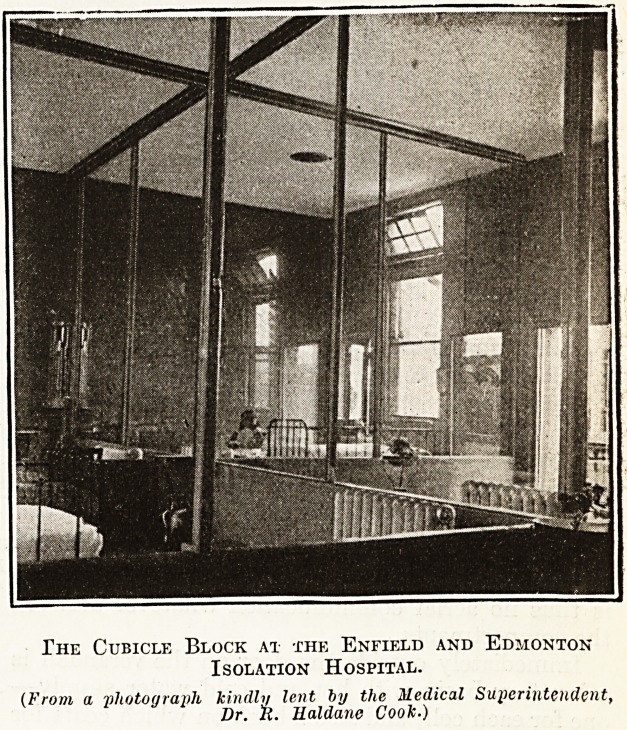


**Figure f2:**